# Counseling Challenges with Variants of Uncertain Significance and Incidental Findings in Prenatal Genetic Screening and Diagnosis

**DOI:** 10.3390/jcm3031018

**Published:** 2014-09-12

**Authors:** Lauren Westerfield, Sandra Darilek, Ignatia B. van den Veyver

**Affiliations:** 1 Department of Molecular and Human Genetics, Baylor College of Medicine, One Baylor Plaza, Houston, TX 77030, USA; E-Mails: lewester@texaschildrens.org (L.W.); sdarilek@bcm.edu (S.D.); 2 Texas Children’s Pavilion for Women, Texas Children’s Hospital, Houston, TX 77030, USA; 3 Department of Obstetrics and Gynecology, Baylor College of Medicine, One Baylor Plaza, Houston, TX 77030, USA

**Keywords:** prenatal, genetic testing, incidental findings, variants of uncertain significance, genetic counseling

## Abstract

Prenatal genetic screening and testing provides prospective parents information about the health of their fetus. It is offered to find or address an increased risk for chromosomal abnormalities or other genetic conditions in the fetus or to identify the cause of fetal structural abnormalities detected by prenatal imaging. Genome-wide tests, such as the already widely-used chromosomal microarray analysis and emerging diagnostic whole exome and whole genome sequencing, have improved the ability to detect clinically significant findings, but have also increased the chance of detecting incidental findings and variants of uncertain significance. There is an extensive ongoing discussion about optimal strategies for diagnostic laboratories to report such findings and for providers to communicate them with patients. While consensus opinions and guidelines are beginning to appear, they often exclude the prenatal setting, due to its unique set of challenging considerations. These include more limited knowledge of the impact of genetic variants when prospectively detected in an ongoing pregnancy, the absence or limitations of detecting clinically recognizable phenotypes at the time of testing and the different decision-making processes that will ensue from testing. In this review, we examine these challenges within the medical ethical framework unique to prenatal care.

## 1. Introduction

New developments in genetic testing and their adoption into clinical practice are occurring at an unprecedented pace. This is not limited to clinical genetic testing in adult and pediatric medicine, but is also extending into prenatal care. The ever-increasing amount of information that can be obtained through these new tests has provoked reflection and debate about whether to report all findings identified by a genetic test or to limit result reporting to certain classes of findings, tailored to the indication for testing and the predicted health consequences of the findings. While recommendations on how to report and counsel about variants of uncertain significance (VOUS) and incidental findings (IF) are becoming available for adult and pediatric healthcare settings, there are currently no official guidelines for prenatal genetic diagnosis and screening, although some publications discussing and addressing these controversial issues are emerging [1]. Because prenatal genetic testing carries with it its own unique set of considerations, discussion and recommendations on how and when to report IFs and VOUS and how to counsel about their implications for the health of the fetus and family are urgently needed. In this paper we will provide a definition of IFs and VOUS and examples of such findings identified through prenatal testing. We will discuss the testing considerations unique to the prenatal setting and examine the challenges and issues that need to be taken into consideration when deciding whether, how and by whom these types of findings should be reported to individuals/couples undergoing prenatal genetic testing.

## 2. Definitions and Examples of Incidental Findings and Variants of Uncertain Significance

In order to better facilitate a discussion about the challenges and impacts of IFs and VOUS, we first need to define these terms and how they overlap. An incidental finding is an observation or result of potential clinical significance that is unexpectedly discovered in a patient, but which is unrelated to the purpose of the diagnostic study or test [2] or that is discovered in a healthy subject. It is important to recognize that IFs are not limited to the field of genetics, but occur in all fields of healthcare and diagnostics. IFs can also occur in the research setting, and it has been noted that as many as 20% of magnetic resonance imaging (MRI) scans performed for research purposes show an IF, with approximately 2% of them requiring urgent medical attention [3,4]. IFs can be categorized based on their clinical utility. They typically fall into one of three categories [5]: actionable (whether immediately or remotely), clinically relevant, but not actionable, and of uncertain significance. An actionable IF is one for which it is well known that a therapeutic or preventive measure exists that can significantly benefit the health of the individual in whom it was discovered. A clinically relevant, but not actionable, finding could, for example, be a carrier status for an autosomal recessive condition for which carrier screening is recommended [6,7,8,9,10]. A variant of uncertain significance is a finding that cannot be unequivocally classified as clinically significant or benign [2]. This could be because there is known variable expressivity or incomplete penetrance for the finding or because the finding is novel and has only very rarely or never been seen before. A VOUS may affect a gene that is relevant to the indication for testing or may be incidental in an unrelated gene. Thus, IFs and VOUS can overlap, creating additional complexity and clinical counseling and management challenges.

These concepts can most easily be illustrated through some scenarios, all derived from real clinical experiences. The first could be one where a chromosomal microarray analysis (CMA) is performed on DNA from amniotic fluid obtained through amniocentesis for a prenatal ultrasound finding of cleft lip and palate. The result shows two copy number variants (CNVs). One is a small copy number loss affecting a gene with a role in palatal development, inherited from the unaffected mother. This CNV is classified as a VOUS, because it is novel and the family history is consistent with possible reduced penetrance. The second is an inherited loss of one copy of a causative gene for an autosomal recessive disorder, indicating carrier status for this disorder. This is primarily an IF, as it is unrelated to and does not explain the cleft palate. However, because it is unknown if there are mutations on the other allele and the copy number loss of this gene has not been previously described, it is also a VOUS. This type of VOUS is of temporary uncertainty, because if mutation analysis of the second allele is performed and no mutations are identified, the genetic counseling can be reassuring, but the degree of residual risk will greatly depend on the population frequency of the condition. Uncertainty can also be temporary, because a CNV that is initially classified as a VOUS may be reclassified to either benign or significant as experience and scientific knowledge about the CNV grow over time. Perhaps the greatest uncertainty occurs when a finding is both an IF and a VOUS of more permanent uncertainty. For example, a patient has a prenatal CMA analysis for advanced maternal age or increased risk of Down syndrome on serum screening, and a deletion is detected in the 16p11.2 proximal region inherited from an apparently healthy father. This deletion has been associated with an increased risk for autism and developmental delay, but with reduced penetrance [11]. In this example, the uncertainty is permanent, as it is inherent to the detected CNV, although more recent studies have indicated that counseling may potentially be more refined in the future, as the presence of second hits at other loci in some cases could further inform risk [12,13].

## 3. Impact of Evolving Technologies and the Scope of the Challenge

### 3.1. Historical Perspective

Detecting IFs and VOUS is not a new problem to the field of prenatal diagnosis. For example, prenatal sonographic imaging can detect subtle changes, such as mildly enlarged lateral ventricles. Recent meta-analysis suggests that this may be associated with an overall 7.9% risk for later neurodevelopmental delay [14], but while this is a useful number for counseling, the actual prognosis for an individual affected fetus cannot be predicted with certainty. Standard karyotype analysis, which has been available for prenatal diagnosis since the 1970s to investigate an increased risk for Down syndrome or other trisomy, because of maternal age or the results from standard maternal serum screening, may also incidentally reveal a marker chromosome or sex-linked aneuploidy, such as 45,X (Turner syndrome) or 47,XXY (Klinefelter syndrome). Since the goal of the performing a karyotype in this scenario is to determine whether a fetus has Down syndrome, the discovered sex chromosome abnormalities are IFs. In the early years of prenatal karyotyping, their prognosis, when incidentally discovered, was more uncertain than what is currently known [15].

### 3.2. Incidental Findings and Variants of Uncertain Significance in Chromosomal Microarray Analysis

Detection of IFs and VOUS has, however, become more frequent and more complex with the emergence of genetic tests that include a more detailed comprehensive analysis of the fetal genome. One such test is CMA, which allows genome-wide detection of aneuploidy and unbalanced chromosomal abnormalities, such as small deletions and duplications at a resolution that is higher than that of a karyotype. CMA has been the recommended test for the pediatric and adult population with developmental disabilities and birth defects since 2010 [16], has gradually become more commonly used in addition to karyotyping for prenatal diagnosis [17] and was recently recommended as the first-line genetic test when a prenatal diagnostic procedure is performed for sonographically detected fetal structural anomalies [18].

In 2012, a multicenter trial funded by the National Institutes of Health (NIH) in the United States prospectively evaluated the performance of CMA compared to traditional karyotyping in over 4000 pregnancies [19]. This study showed that prenatal CMA identified all clinically significant aneuploidies and unbalanced translocations found by karyotype analysis. In addition, CMA also identified a known pathogenic chromosomal abnormality with the potential for clinical significance in 1.6% of fetuses with indications, such as advanced maternal age or abnormal maternal serum screening results for Down syndrome, and in 6% of fetuses with structural abnormalities who had a prior normal karyotype [19]. Following the publication of this data, the American College of Obstetricians and Gynecologists (ACOG) and the Society for Maternal-Fetal Medicine (SMFM) published a joint committee opinion in December, 2013, on the use of CMA for prenatal diagnosis [18]. In this opinion, they recommend that in cases where invasive prenatal diagnosis is being pursued because one or more structural abnormalities are found by prenatal ultrasound, CMA should replace karyotyping as the first-line diagnostic test and that in cases where a pregnant woman elects to undergo invasive prenatal testing for other indications, CMA can be offered, and its use should not be restricted to women 35 years or older [18]. They further state that there is a critical need for expert pre- and post-test genetic counseling to address potentially complex results and VOUS [18]. Although CMA was already widely offered prior to December, 2013 [17,20,21,22], it is expected that with the publication of these recommendations, its use will increase as the first-tier prenatal diagnostic test for pregnancies when a diagnostic procedure, such as amniocentesis or chorionic villus sampling, is performed, because of an increased risk for fetal chromosomal abnormalities. The NIH-sponsored multicenter trial showed that variants of uncertain significance can be found in 3.4% of tested pregnancies, but 1.8% of these were classified by a clinical oversight committee as likely benign. This is similar to other data, including our own experience of VOUS in 1.6% of prenatal CMA [20] and a prospective cohort study and systematic review and meta-analysis with a VOUS detection rate of up to 2% for prenatal CMA [17].

### 3.3. Incidental Findings and Variants of Unknown Significance in Diagnostic Next Generation Sequencing

More recently, new genetic tests have been developed that use high-throughput next generation sequencing (NGS) technology to achieve simultaneous sequencing and mutation analysis of multiple genes in a single assay. This has resulted in the development of research and diagnostic tests that analyze disease-specific gene panels, all sequenceable exons (whole exome sequencing or WES) or the entire genome (whole genome sequencing or WGS). Diagnostic WES or WGS with its goal of finding mutations in single genes are currently not routinely available for testing ongoing pregnancies, outside of research. However, we predict that this is will occur soon, and it has already been employed to study the causes of birth defects that result in stillbirth or poor neonatal outcome in a prior pregnancy and on a research basis [5,23,24]. Once WES or NGS gene panels become available for prenatal genetic diagnosis, they will expand the options parents have available beyond CMA, karyotype and analysis of a few single genes when there are abnormal ultrasound findings or a family history suggestive of an unknown genetic condition, to testing for hundreds of genetic disorders simultaneously. While this promises to provide parents with more information about the health of their fetus, with an initial estimation of up to a 10% incremental benefit with WES [23], the risk for identifying IFs and VOUS also increases. In fact, some predict that with such tests, the likelihood of finding a VOUS is actually higher than that of finding a mutation that explains the phenotype. Because these high-throughput genetic diagnostic methods typically require analyzing parents to aid with the interpretation of findings in an affected child or in the case of prenatal diagnosis, the unborn fetus, it is also possible that IFs in parental samples will be discovered, adding additional complexity to result reporting and counseling. Because of the potential incremental benefit, we predict that prenatal diagnostic use of WES and NGS panels will increase in the near future. Importantly, prenatal care providers and genetic counselors are already being asked to counsel families about implications for an ongoing or planned pregnancy of results from diagnostic WES performed for a family member. In at least 1% or more instances [25], such results will include reported IFs and VOUS, but their actual incidence depends on how the reporting of WES results is approached by the diagnostic laboratory and what amount of that information is then shared by the healthcare providers with the individuals whose DNA was sequenced or with the parents in case of a minor. These circumstances create very challenging prenatal genetic counseling circumstances; an example would be a VOUS in a plausible candidate gene detected in a previous severely affected child and parents then requesting prenatal or preimplantation genetic diagnosis for this VOUS to avoid recurrence.

### 3.4. Incidental Findings and Variants of Unknown Significance in Non-Invasive Prenatal Screening

Non-invasive prenatal screening (NIPS) is currently commercially offered to screen with very high sensitivity and specificity whether a pregnancy is affected with trisomy 21, trisomy 13 or trisomy 18 or a sex chromosome aneuploidy and to determine the gender of the fetus [26,27,28]. Although individual methods vary, all approaches employ NGS on cell-free DNA isolated from maternal plasma, about 10% of which is derived from the placenta and, thus, reflects the fetal genome [27]. A quantitative analysis of the sequence for each chromosome of interest is then derived to determine whether the fetus has aneuploidy for the analyzed chromosomes. More recently, laboratories have also begun to investigate deletions and duplications using this approach [29,30,31]. NIPS is a screening tool, and false positive and false negative results are therefore expected; but, they initially came as a surprise to some providers and patients, creating challenging counseling situations. Since then, it has also become clear that the follow-up confirmatory studies can reveal unexpected findings in both the maternal and fetal genome that are unrelated to the aneuploidy for which the NIPS showed an increased risk. Reported IFs range from fetal or maternal deletions and duplications or mosaic sex chromosome aneuploidy in the mother or fetus, presenting as aneuploidy risk on NIPS, to mosaicism and uniparental disomy to abnormal results because of the presence of cell-free DNA originating from an undiagnosed maternal tumor [32,33,34,35,36,37,38]. Although providers have become aware of these possibilities, little guidance currently exists on how to inform patients about them in pre- and post-test counseling.

## 4. Special Counseling Considerations in Prenatal Testing

### 4.1. Unique Clinical Circumstances of Prenatal Genetic Testing

Several aspects of prenatal genetic testing set it apart from that in the pediatric and adult population, where clinical genetic testing is typically undertaken to provide a diagnosis and inform medical management and treatment options for individuals with an observable phenotype. In contrast, the goal and purpose of prenatal genetic testing is often the desire to gain information about the health of a fetus who may be at increased risk for a chromosomal or other genetic condition, but has no known phenotype or clinically detected abnormalities, or has an incompletely defined phenotype, because of the limitations of prenatal imaging and prenatal dysmorphology. In many cases, the test is performed to rule out rather than confirm a particular diagnosis or find a diagnosis for an unresolved phenotype and provide reassurance, rather than to confirm a clinically suspected diagnosis. Another goal of prenatal genetic testing can be to gain information that can inform decisions regarding the continuation or termination of an affected pregnancy, to help plan for optimal prenatal, perinatal or neonatal management and/or to prepare prospective parents for a poor neonatal outcome. In the majority of prenatally-diagnosed genetic conditions, there is very little, if any, treatment available during the prenatal period. Thus, a unique aspect to prenatal diagnosis is that individuals undergoing this testing have the ability to use this information to make decisions about the continuation of a pregnancy [39]. In some cases, such as for ongoing pregnancies with a predicted lethal outcome for the infant after birth, the information is used by parents and healthcare providers to inform a choice between aggressive perinatal and neonatal management or comfort care. These prenatal and perinatal care decisions affect not only the wellbeing of the fetus, but can have important consequences for the physical, mental and social wellbeing of the parents and the immediate and extended family members [1].

### 4.2. Unique Aspects of Prenatally Detected VOUS

Many factors need to be considered in interpretation and counseling about the potential clinical consequences of detected VOUS. First, when a VOUS is detected in a pediatric or adult patient, laboratory directors and clinicians use information about a patient’s known phenotype to help with interpreting the implications of the discovered VOUS for the health of the tested individual. However, prenatally, the phenotypic information is often incomplete and sometimes inaccurate, making the interpretation and, consequently, genetic counseling about a new prenatally discovered VOUS more challenging. Second, for CNVs or other genetic changes that have been associated with a clinical phenotype, one has to take into account that the clinical information is almost exclusively derived from postnatal data of the testing performed to arrive at a diagnosis in affected individuals. Furthermore, the literature on the effects of deletions and duplications or other mutations is also potentially biased towards cases at the more severe end of the phenotypic spectrum, since they are more likely to come to clinical attention and the findings are more likely to be published. For many of these, there are no good data on the predicted prognosis and outcome when detected prenatally or in healthy individuals. Such data are however continuously being collected in ongoing long-term follow-up studies and data repositories, such as ClinVar [40], the Patient Crossroads’ Prenatal Array database [41] or the ClinGen resource at the International Collaboration for Clinical genomics (ICCG) [42] and DECIPHER (Database of Chromosomal Imbalance and Phenotype in Humans Using Ensembl Resources) [43]. This leads to a third challenge with interpreting VOUS, which is that the knowledge of the pathogenic significance of certain VOUS is adjusted over time due to evolving knowledge about these variants. The fourth consideration is that if a CNV is found in a fetus with an abnormality detected by ultrasound, it may be suggestive of the pathogenicity of the CNV variant; but, it is also possible that the finding is coincidental, and causality is not always easy to confirm. Fifth, benign CNVs, as well as VOUS are often inherited from an apparently normal parent. While this is reassuring in many cases, in light of growing evidence of incomplete penetrance and variable expressivity of some VOUS, the inheritance of a VOUS from a parent may not be as helpful in counseling as was previously thought [39].

### 4.3. Unique Aspects of Prenatally Detected IFs

When IFs are detected prenatally, there may be consequences for the fetus, as well as for the parents and other family members. This can create significant counseling difficulties. For example, an IF could be a genetic change that inevitably leads or predisposes to an inherited adult-onset disorder, but that has no implications for the immediate prognosis of the fetus or later, the child, and for which early surveillance may not affect the outcome. Guidelines exist that discourage specific testing for such adult-onset disorders in children who cannot themselves consent for such testing, because of the potential psychological harm to the child from the knowledge of a presymptomatic mutation that causes a severe adult-onset disorder, such as, for example, early-onset Alzheimer’s or Huntington’s disease or a predisposition to breast cancer. However, it is less clear how to handle such IFs when they are discovered incidentally through genome-wide genetic tests. It can be argued that they should not be sought for and/or reported during prenatal diagnosis. However, the context of the entire family has to be considered in such decisions. For example, if a maternally inherited mutation that predisposes to breast cancer is found in the fetus, this information could lead to increased surveillance of the mother and potentially life-saving early detection, and it could lead to the discovery of other family members at risk. From this perspective, withholding this information could cause harm. Other types of IFs could be relevant to certain traits, but are not disease-causing. The best-known example is the fetal gender, which is not a new issue that emerged with the newer genomic tests and, in some societies, can lead to terminating pregnancies of the undesired gender.

### 4.4. Patient Perception and Counseling Considerations

There is very limited data on how prospective parents perceive information about IFs or VOUS in genetic testing results and on the impact it has on them and their families. In a qualitative study, 23 women were interviewed about how they experienced and responded to receiving abnormal results from prenatal CMA performed in a research setting [44]. Repeated elements that characterized the concerns and experience of participants included “uncertainty”, “unquantifiable risk” and “toxic knowledge”. Some women found that variable expressivity and, hence, the lack of a precise risk estimate for a medical issue in their future child from a VOUS made it difficult to process information about how their child might be affected by the genetic finding. This was exacerbated by the lack of knowledge about the health and development of the child that would have been available if testing had been performed postnatally. Some of the participants, when told that their child may or may not have a problem of variable severity, expressed regret about having received this information. Women who continued their pregnancies reported that having the CMA results changed how they experienced being pregnant and watching their child develop. Although this study was limited by its small size and by the fact that it could not be determined if women who volunteered to participate differed significantly in their experience from those who declined, it provides insight into what some women experience when they get results from more comprehensive prenatal testing that include IFs or VOUS. Nevertheless, Fernandez *et al*. explored the attitudes of parents with respect to the return of targeted and incidental results in a pediatric research setting and found that many parents have a strong desire to receive a broad range of results [45]. In this study, 86% of parents indicated that they wanted to receive results indicating an IF, 83% wanted results predicting susceptibility to even untreatable fatal conditions, 87% wanted results for multiple types of conditions and 70% wanted results with an uncertain impact. In another study that investigated the use of single nucleotide polymorphism (SNP) arrays for prenatal diagnosis, it was found that 89% of parents wanted results that probably would have an adverse health affect in infancy and childhood and 55% wanted results that probably would have an adverse healthy affect in adulthood [46].

### 4.5. Guidance from an Ethical Framework?

Attempts to determine whether providers have the ethical responsibility to report all, none or a subset of such IFs and VOUS tend to consider core values of medical ethics: autonomy, non-maleficence and beneficence. The question to be answered is whether an individual’s autonomy, in this case, the right to choose a prenatal test that may return results of uncertain significance or IFs unrelated to the indication for testing and receive all this information, outweigh the potential harm that may be caused by these findings on the future child, on the pregnant woman and how she views her pregnancy after this result or on other family members (non-maleficence). Some argue that a woman’s autonomy, in the context of informed consent, is the most important ethical consideration [47]. They acknowledge that maternal anxiety is an expected response to abnormal test results or VOUS and not a reason to withhold testing, but that providers should take steps to minimize patient distress. There is an ongoing debate as to whether such information obtained through comprehensive analysis of the fetal genome should be withheld, because it could result in increased terminations for “insignificant” DNA changes and may undermine the aim of prenatal screening “to help couples have healthy babies”, thereby causing harm [48]. However, McGillivray *et al*. state that the role of prenatal diagnosis is “to give women the opportunity for informed choice about their pregnancies and the children they have” and that all prenatal testing results, including IFs or VOUS, are information that a woman should be able to consider. While uncertainty may cause distress, parents could justifiably consider it in decisions about a pregnancy or the needed developmental assessment and care for their future child [47]. From that perspective, there is potential that withholding this information could cause harm to the woman (parents) and child.

Finally, knowledge about the medical implications of genetic variants is rapidly increasing alongside advances in medical care, and it cannot be predicted what the potential future benefit may be of knowing about a particular variant that was initially of uncertain significance. Considering this, the temporary emotional distress must be weighed against the potential future benefit of knowing this information. Thus, it is uncertain if a pregnant patient’s autonomy to elect not to receive information on IFs or VOUS on the basis of wanting to avoid anxiety always supersedes beneficence obligations and the duty to warn [1]. Guidelines on how to address these challenging questions are needed, but developing them will be difficult and complex. Equally challenging is that patients need to have access to the necessary expert pre- and post-test genetic counseling, so that distress can be minimized and patients can be supported to make well-informed decisions with the information available to them. This is best done by providers, such as genetic counselors or medical geneticists, who are familiar with the various testing modalities and the categories of results they return and who are trained in effective strategies to communicate difficult results, assist patients in coping with the health implications of these results and support them in their informed decisions. Unfortunately, as access to genome-wide testing increases, there may not be enough subspecialty-trained professionals, and thus, there is an urgent need to develop alternative counseling strategies and enhance the training in genetics of medical professionals from other disciplines.

## 5. Need for Practice Guidelines

Currently, there are no established guidelines that specifically address how IFs and/or VOUS should be handled in the field of prenatal genetics. This leaves the burden on individual diagnostic laboratories and clinical providers to determine which results to disclose and how best to communicate them, creating a lack of consistency. Guidelines from the ACMG for reporting CMA results in the postnatal setting are available [2] and are often applied to prenatal cases. However, postnatally, patients undergo testing after the onset of symptoms in an attempt to explain what is already present, while with prenatal CMA the indication is often to reassure that a significant finding is absent. This can lead to greater uncertainty, since one cannot differentiate an asymptomatic from a pre-symptomatic fetus. The ACMG recognized that it is not possible to construct a diagnostic CMA platform that avoids loci associated with certain categories of IFs, such as loci associated with recessive carrier status, predisposition to cancer, detection of a presymptomatic stage of a condition or detection of a condition with an unrecognized clinical presentation [2]. The recommendations put forth were mostly focused on well-characterized IFs and include: (1) considering the disclosure of the copy number loss of recessive disease loci if the disease was well categorized and the carrier frequency was relatively high or the clinical features were consistent with the patient’s reason for referral; (2) in the case of presymptomatic or clinically undetected conditions, disclosing the results in order to facilitate access to care, but individual laboratories could choose not to disclose certain results for adult-onset conditions; and (3) carefully considering deletions involving known or putative tumor suppressor genes, but, in general, discussing the findings of well-characterized ones that have management and surveillance implications, while avoiding speculation about others.

Other areas of medicine and research have addressed IFs before the era of genomic testing, for example IFs in medical and research imaging, and have generally adopted the view that they should be disclosed and confirmed, as not disclosing could potentially result in harm [49]. In 2013, the ACMG also published recommendations for the reporting of IFs detected by WES performed in pediatric and adult populations by diagnostic laboratories to the requesting healthcare providers, but specifically excluded prenatal diagnostic settings and specified that these recommendations only apply to the obligations of the laboratory and that the decision to communicate IFs to patients is outside the responsibility of the laboratory and should be decided by the care provider within the provider-patient relationship. The guidelines included a list of 56 genes for which there was consensus that pathogenic mutations should be reported [25]. Recent updates to the policy recommend that patients be allowed the option to opt-out of such results [50]. This list represents well-described, medically actionable results with a high probability of an adverse medical outcome that is potentially preventable by medical surveillance or treatment that would not be initiated without the diagnostic result [50]. It is also recommended that VOUS, variants associated with low or unknown penetrance and variants associated with disorders not currently amenable to intervention should not be reported as IFs. These guidelines have stirred controversy, resulting in additional clarification regarding their scope and goals [51] and will likely be adjusted and updated as experience with clinical WES grows. Alternate approaches for addressing patient preferences about what type of incidental information to disclose have been suggested. These include a two- or three-tiered reporting system, already in use by some diagnostic WES laboratories: they could range from a focused report targeted to findings that are directly or potentially related to the phenotype of interest; a more expanded report that includes the IFs recommended in the ACMG guidelines and, maybe, other IFs or VOUS that are highly likely to be pathogenic or medically actionable; to a comprehensive report that includes all of the above ,as well as additional VOUS for which potential pathogenicity is less well established. The optimal approach may well have to be individualized and would require good communication between the patient and his/her clinical care provider; and between the provider and the laboratory. One foreseeable limitation is that information could be withheld that may have clinical relevance in the child’s future, as variants are reclassified or additional phenotypic features emerge with age.

## 6. Conclusions

With the emerging integration of genomic testing in prenatal diagnosis comes the responsibility for healthcare professionals to manage IFs and VOUS that are inherent to these techniques. The reporting and counseling challenges that accompany them are complex and probably best handled by genetics professionals who are more accustomed to this type of information. However, these diagnostic tests are being ordered more frequently outside the realm of genetics, and other healthcare providers may need additional training, as well as guidelines that they can follow to help them convey this information to their patients. In a survey of 40 clinicians who had ordered CMA, many non-genetics professionals did not feel equipped to interpret the results for patients, and despite clinical guidelines recommending informed consent, many clinicians did not consider it pertinent to discuss the potential for CMA to reveal incidental information, such as biological parentage or predisposition to adult-onset disease. They also reported a greater likelihood to disclose an incidental finding that they viewed as actionable (colon cancer predisposition) *versus* non-actionable (Alzheimer’s disease) [18]. Clear guidelines regarding how the informed consent and results disclosure process should handle incidental findings and VOUS would be useful, not only to genetic counselors and geneticists, but also to non-genetics clinicians, who will continue to order genome-wide tests with increasing frequency as the technology expands.
